# CpG oligodeoxynucleotides attenuate RORt-mediated Th17 response by restoring histone deacetylase-2 in cigarette smoke-exposure asthma

**DOI:** 10.1186/s13578-021-00607-3

**Published:** 2021-05-20

**Authors:** Hongtao Li, Qimei Ye, Yusen Lin, Xuena Yang, Xiaoling Zou, Hailing Yang, Wenbin Wu, Ping Meng, Tiantuo Zhang

**Affiliations:** Department of Pulmonary and Critical Care Medicine, The Third Affiliated Hospital of Sun Yat-sen University, Institute of Respiratory Diseases of Sun Yat-Sen University, Guangzhou, Peoples Republic of China

**Keywords:** Asthma, Histone deacetylase 2, Retinoid-related orphan nuclear receptor t, Th17 polarization, Corticosteroid insensitive

## Abstract

**Background:**

Cigarette smoke (CS) exposure increases corticosteroid insensitive asthma related to increased Th17 phenotype, and new treatment strategies are needed for CS-associated asthma. Histone deacetylase 2 (HDAC2), found in the airway epithelium, is critical for ameliorating glucocorticoids insensitivity. We recently demonstrated the anti-inflammatory effects of CpG oligodeoxynucleotides (CpG-ODNs) on CS-exposure asthma. However, the effects of CpG-ODNs on HDAC2 expression and enzymatic activity remain unclear. This study aimed to assess whether CpG-ODNs protect against excessive Th17 immune responses in CS-induced asthma through HDAC2-dependent mechanisms and compared their effects with those of corticosteroids.

**Methods:**

The effects of CpG-ODNs alone and in combination with budesonide (BUD) on airway inflammation and Th2/Th17-related airway immune responses were determined using an *in vivo* model of CS-induced asthma and in cultured bronchial epithelial (HBE) cells administered ovalbumin (OVA) and/or cigarette smoke extract (CSE). HDAC2 and retinoid-related orphan nuclear receptor t (RORt) expression were also assessed in mouse lung specimens and HBE cells.

**Results:**

CpG-ODNs and BUD synergistically attenuated CS exposure asthmatic responses *in vivo* by modulating the influx of eosinophils and neutrophils, airway remodeling, Th2/Th17 associated cytokine and chemokine production, and airway hyperresponsiveness and blocking RORt-mediated Th17 inflammation through induced HDAC2 expression/activity. *In vitro*, CpG-ODNs synergized with BUD to inhibit Th17 cytokine production in OVA- and CSE-challenged HBE cells while suppressing RORt and increasing epithelial HDAC2 expression/activity.

**Conclusions:**

CpG-ODNs reversed CS-induced HDAC2 downregulation and enhanced the sensitivity of CS-exposed asthmatic mice and CSE-induced HBE cells to glucocorticoid treatment. This effect may be associated with HDAC2 restoration via RORt/IL-17 pathway regulation, suggesting that CpG-ODNs are potential corticosteroid-sparing agents for use in CS-induced asthma with Th17-biased immune conditions.

**Supplementary Information:**

The online version contains supplementary material available at 10.1186/s13578-021-00607-3.

## Introduction

Asthma is an increasingly prevalent respiratory ailment that affects at least 300 million individuals worldwide, with approximately 345,000 deaths annually [1]. Approximately 25% of the adult population in developed nations smokes, and a survey of asthma patients suggested that the rate of smoking among patients mirrored that of the general population [2]. Cigarette smoke (CS) directly and passively increases asthma susceptibility, decreases quality of life, enhances symptom severity, and exacerbates attack frequency [3]. According to several clinical studies, asthmatic smokers show poorer responses to treatment with steroids than nonsmokers [4, 5]. After exposure to CS, some cases of asthma progress to uncontrolled asthma, which is also called severe asthma.

Although inhaled corticosteroids (ICSs) are the standard therapeutic option for asthma, individuals show various responses, and most severe asthma patients may be insensitive to steroid-mediated suppression [6]. Asthma is currently considered a heterogeneous ailment, involving Th1, Th2 and Th17 cells [7]. In general, the Th2 immune response, which features eosinophil influx, substantially contributes to the development of allergic airway inflammation. Individuals with mild-to-moderate asthma mostly exhibit this disease type and can be treated with classic therapies, such as ICSs. Moreover, severe asthma is hardly manageable, even with the newest drugs. Severe asthma cases exhibit a mixed Th1/Th2 phenotype comprising a Th17 component, with elevated neutrophil rates or neutrophils plus eosinophils in the lung and sputum [8, 9]. We previously demonstrated that CS exposure asthma was associated Th17 differentiation and budesonide (BUD) had limited effects on neutrophil infiltration in bronchoalveolar lavage fluid (BALF), which suggested CS exposure asthma may be relatively insensitive to glucocorticoids (GCs) [10]. This finding could explain why asthma patients exposed to CS directly or passively may exhibit reduced responsiveness to steroids. Approximately 525% of severe asthma cases show poor symptom control even after the administration of high-dose and/or systemic GCs, which contributes to nearly 50% of all asthma-associated treatment costs [11]. Accordingly, effective drugs are urgently required as mono or adjuvant therapies.

Corticosteroid insensitivity in mixed granulocytic asthma might be due to multiple factors. Classically, the molecular mechanisms of corticosteroid insensitivity mainly include the overexpression of proinflammatory transcription factors, the phosphorylation of GC receptors (GRs), and the loss of histone deacetylase-2 (HDAC2) expression [12]. A study by Li et al. suggested that HDAC2 is required for corticosteroid-associated anti-inflammation [13]. Specifically, GCs decrease inflammatory reactions via HDAC2 recruitment to the promoters of proinflammatory genes, regulating the transcription of these genes [14]. According to several studies, HDAC2 activity is decreased in alveolar macrophages, peripheral blood mononuclear cells, and bronchial biopsies of asthma patients [15,17]. Moreover, studies have also demonstrated that HDAC2 protects against airway inflammation in murine and human epithelial cells. Notably, oxidative stress induced by CS impairs HDAC2 function via ubiquitination-proteasome-dependent degradation, leading to amplification of the inflammatory response and GCs insensitivity *in vitro* and *in vivo* [16, 18,21]. Therefore, GCs insensitivity correlates with HDAC2, suggesting that drugs that restore HDAC2 activity and expression could ameliorate GCs insensitivity.

CpG oligodeoxynucleotides (CpG-ODNs) are unmethylated CpG dinucleotides that mimic the immunostimulatory effects of bacterial DNA and stimulate Toll-like receptor 9 (TLR9). CpG-ODNs have shown beneficial effects in many rodent and primate models of asthma and encouraging preliminary results in clinical studies [22]. These reports suggested that CpG-ODNs induce Th1 responses and limit Th2 responses [23]. Our study and others indicated that CpG-ODNs have both potent preventive and therapeutic immunomodulatory effects on allergic inflammatory diseases [24]. The benefits of CpG-ODNs in protecting against CS-induced airway inflammation are associated with reductions in excessive CS-induced Th2/Th17 immune responses and increased Th1 responses based on our previous study [10], and further investigation of the underlying mechanism is of great interest. Retinoid-related orphan nuclear receptor t (RORt) is a transcription factor that regulates IL-17 A [25]. Despite substantial efforts to understand CpG-ODNs-related anti-inflammation, it remains unclear whether or how CpG-ODNs attenuate the RORt-mediated Th17 response by restoring HDAC2 activity and expression, thereby ameliorating GCs insensitivity.

The current follow-up study investigated the mechanism by which CpG-ODNs regulate HDAC2 expression/activity and modulates subsequent inflammatory responses in a mouse model of CS-related asthma and human bronchial epithelial (HBE) cells. We established a mouse model of ovalbumin (OVA)-induced asthma after CS exposure, as well as *in vitro* cultures of HBE cells exposed to OVA and CSE, and administered CpG-ODNs and BUD to assess the effects of CpG-ODNs on airway inflammation and remodeling, as well as GCs insensitivity associated with HDAC2 in mice co-exposed to chronic CS and OVA. To the best of our knowledge, this was the first study to show CpG-ODNs could restore steroid sensitivity and block RORt-induced upregulation of IL-17 in CS-induced asthma *in vivo*, as well as in CSE-induced HBE cells, possibly through the restoration of HDAC2 levels and activity.

## Materials and methods

### Mice and experimental design

Female specific pathogen free BALB/c mice (six- to seven-weeks old), provided by the Laboratory Animal Center of Southern Medical University, China (No.44,002,100,019,453), were housed under standard laboratory conditions including a 12 h/12 h light-dark cycle and rodent chow and water for 3 days *ad libitum*.

Experimental animals were randomized to seven groups (12 animals/group), including the vehicle control, CS, OVA, OVA/CS, CpG-ODN, BUD and CpG-ODN/BUD groups. The latter 5 groups were sensitized and challenged with OVA. After each challenge, the mice of the latter 4 groups were exposed to CS in ventilated whole-body smoking chambers as previously described [10]. CpG-ODNs and/or BUD were administered intranasally to the last 3 groups half an hour post-OVA challenge as previously described [10]. The vehicle control and CS groups were not sensitized or challenged. Moreover, the vehicle control, CS and model groups were treated with NS as negative or positive controls. The chronic CS-exposure asthmatic murine model was established as described in a previous report, with minor modifications [9]. Details are provided in the online supplementary material. A schematic diagram of the CS exposure asthmatic murine model and treatments is depicted in Additional file 1: Fig. S1.

### Laboratory measurements of the murine model

Additional details of BALF sampling, the quantification of cytokines in BALF, tissue histology, immunohistochemistry, immunoblotting, serum IL-17 A and OVA-specific IgE level assessment, fluorescence microscopy, quantitative reverse transcription polymerase chain reaction (qRT-PCR), flow-cytometric analysis, and airway hyperresponsiveness (AHR) measurement are provided in the Additional file 1.

### HDAC2 activity

Nuclear HDAC2 activity in the nuclear extract was measured with an HDAC2 IP & Activity Assay Kit (BioVision Mountainview, CA, USA) according to the manufacturers instructions. We analyzed the fluorophore and an excitation wavelength of 360 nm and an emission wavelength of 460 nm with a fluorescence plate reader.

### CSE preparation

CSE preparation was performed as previous described, with minor modifications [26]. Briefly, one cigarette (each cigarette: nicotine, 1.0 mg; tar oil, 10 mg; carbon monoxide, 13 mg; Tobacco Hunan Industrial Corporation, China) was combusted, and the smoke was passed through 10 mL of serum-free culture medium at a rate of 5 min/cigarette. The pH of the medium was adjusted to 7.4 and diluted as indicated with culture medium. Freshly prepared CSE was used within 30 min.

### Cell culture

HBE cells, provided by the American Type Culture Collection (ATCC PCS-300-010), underwent culture in RPMI 1640 containing 10% fetal bovine serum (FBS). Then, the cells were administered 2.5, 5 and 10% CSE, respectively, for 6 h, 12 h, 24 h, 48 or 72 h for detecting the dose/time effects of CSE on HBE cell proliferation.

### Cytotoxicity assay

The cell viability in response to stimulation with of CSE for different times was examined using a standard 3-(4,5-dimethylthiazol-2-yl)-2,5-diphenyl tetrazolium bromide (MTT) assay [27]. Stimulation with CSE affected the proliferation and viability of HBE cells. A certain dose of CSE could stimulate the proliferation of HBE cells, while increasing doses of CSE induced cytotoxicity, not only inhibiting HBE cells but also inactivating them, which suggested that a specific concentration of CSE reduced the viability of HBE cells. Based on Additional file 1: Fig. S2 (see Additional file 1: Fig. S2), HBE cells exposed to 2.5% CSE for 6 h exhibited stable and nearly natural cell proliferation; therefore, we chose 2.5% CSE to treat HBE cells for 6 h to reduce experimental errors. Treatment doses of CpG-ODNs and BUD were determined in preliminary experiments.

### Cell treatments

HBE cells were similarly divided into seven groups. (1) Vehicle control group: HBE cells were administered phosphate buffer saline (PBS), followed by PBS treatment. (2) CSE group: HBE cells were administered 2.5% CSE, followed by PBS treatment. (3) OVA group: HBE cells were administered 1 g/ml OVA, followed by PBS treatment. (4) CSE/OVA group: HBE cells were administered 1 g/ml OVA and 2.5% CSE, followed by PBS treatment. (5) CpG-ODN group: HBE cells were administered 1 g/ml OVA and 2.5% CSE, followed by 510^6^ M CpG-ODN treatment. (6) BUD group: HBE cells were administered 1 g/ml OVA and 2.5% CSE, followed by 10^8^ M BUD treatment. (7) CpG-ODN/BUD group: HBE cells were administered 1 g/ml OVA and 2.5% CSE, followed by 10^8^ M BUD and 510^6^ M CpG-ODN treatment.

### Laboratory measurements of HBE cells

The amounts of IL-5, IL-13 (Th2 cytokines) and IL-17 A (Th17 cytokine) were assessed by specific enzyme-linked immunosorbent assay (ELISA) kits (Bioss Inc., China) as directed by the manufacturer. The relative mRNA levels of cytokines in HBE cells were assessed by qRT-PCR. The protein expression levels of IL-17 A (Invitrogen, USA), HDAC2 (Invitrogen, USA) and RORt (Invitrogen, USA) were detected by Western blotting and immunofluorescence analysis. Flow cytometry antibodies detecting HDAC2, RORt and IL-17 A were provided by Abcam (US). Flow cytometry was carried out on a BD Calibur instrument (BD, USA).

### Statistical analysis

The data are presented as the meanstandard deviation (SD), and were assessed by one-way analysis of variance (ANOVA) for multiple groups, with post-hoc Tukeys multiple comparison test. GraphPad Prism 6.0 (GraphPad Software, USA) was employed for data analysis, and *p*<0.05 indicated statistical significance.

## Results

### Suppression of combined granulocyte inflammation, airway structural remodeling, and AHR by CpG-ODNs plus BUD in chronic CS-exposed asthmatic mice

**Fig. 1:**
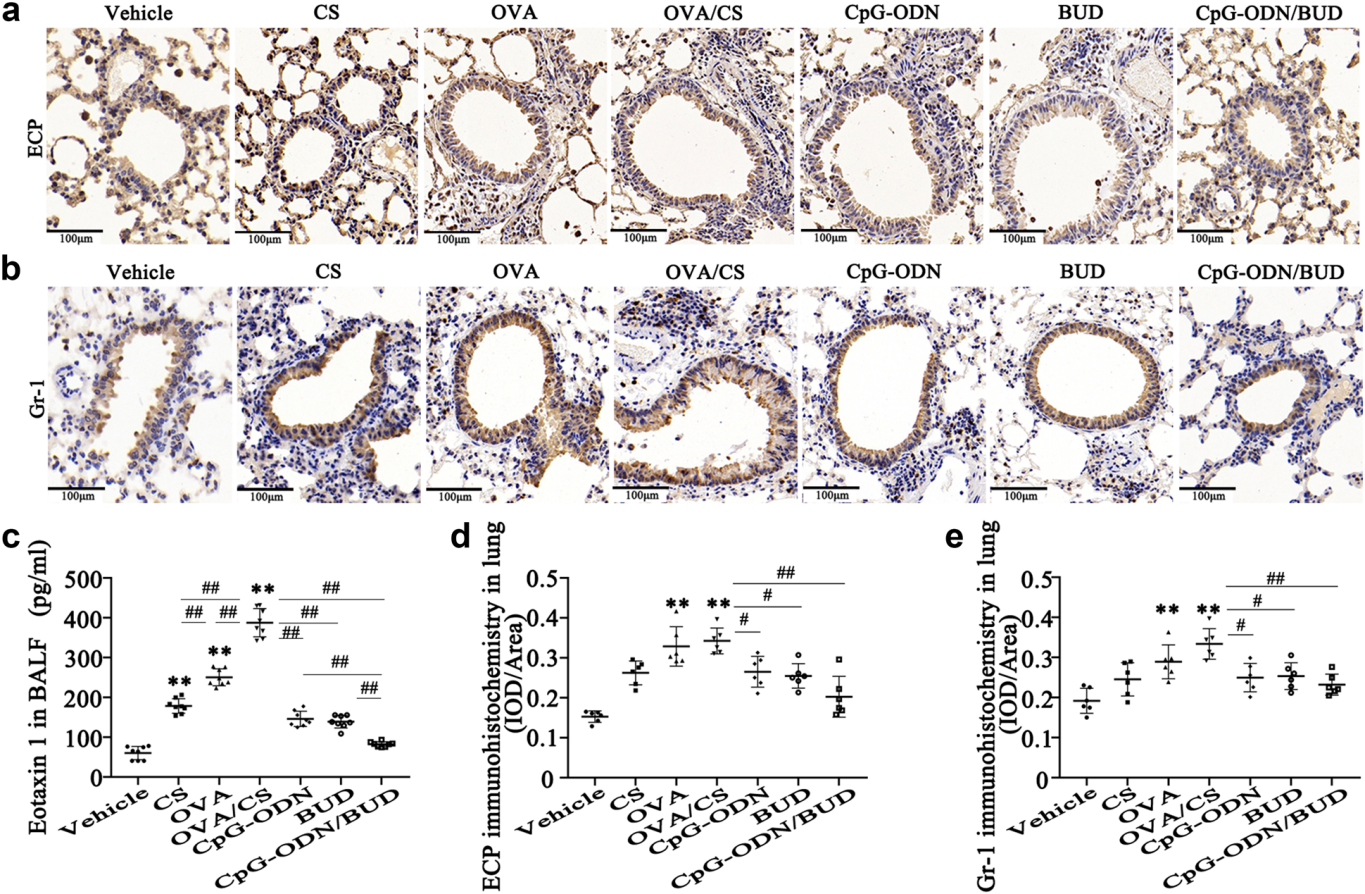
CpG-ODN and BUD alleviate airway inflammation in the lung tissue in mice after OVA-challenge and CS-exposure. Lung tissue samples were histologically assessed 48 h upon final OVA exposure. Representative micrographs of ECP stained specimens (200) (**a**). Representative micrographs of Gr-1 stained samples (200) (**b**). Eotaxin 1 in BALF was assessed by ELISA (**c**). ECP-positivity rate was assessed as Gr-1-positive area/total bronchiole area (**d**). Gr-1-positivity rate was derived as Gr-1-positive area/total bronchiole area (**e**). Statistical significance denoted:^*, #^*p*<0.05, ^**,##^*p*<0.01. *versus the vehicle control group; # versus the indicated group

Histological data showed that lung specimens from OVA/CS mice had substantial peribronchial and perivascular connective tissues (Additional file 1: Fig. S3a), multiple airway goblet cells containing mucus (Additional file 1: Fig. S3b, d), and peribronchial collagen deposition (Additional file 1: Fig. S3c, e). A combined granulocyte (neutrophil and eosinophil) inflammatory phenotype was confirmed as indicated by elevated Gr-1 (neutrophil-specific marker; Fig. 1b, e) and ECP (eosinophil-specific marker, Fig. 1a, d) immunohistochemical signals in the lungs, as well as marked expression of eotaxin 1 in BALF (Fig. 1c), which facilitates the recruitment of eosinophils and neutrophils [28]. The predominance of airway inflammation associated with a mixture of neutrophils and eosinophils was reduced; airway remodeling factors such as goblet cell hyperplasia and collagen accumulation were also diminished in the airways of CpG-ODNs or BUD treated mice (Additional file 1: Fig. S3) compared with those in CS-exposure asthmatic mice. Meanwhile, combined treatment with CpG-ODNs and BUD caused almost no alterations in mucus hypersecretion, negligible cell infiltration or alternations in airway wall thickness alteration, with suppression of AHR upon methacholine administration in animals with CS-exposure asthma (Fig. 1, Additional file 1: Fig. S3).

### Alterations in Th2/Th17 polarization and reductions in proinflammatory cytokines by CpG-ODNs and BUD in CS-associated asthmatic mice

Th2 markers (IL-5 and IL-13) were induced, while the Th1 marker IFN- was reduced after OVA+CS co-exposure in the mouse model (*p*<0.01; Additional file 1: Fig. S4ce and *p*<0.01; Additional file 1: Fig. S4b). Proinflammatory cytokines (IL-8 and TNF-), TGF-1, and serum anti-OVA IgE were also increased (all *p*<0.01, Additional file 1: Fig. S4a, f, g, h, i). These values changed substantially after treatment with CpG-ODNs (Additional file 1: Fig. S4). BUD also somewhat attenuated the CS associated increase in proinflammatory cytokines and serum anti-OVA IgE. However, we also noted that CpG-ODNs combined with BUD had additive beneficial effects on the modulation of Th1/Th2 homeostasis, proinflammatory cytokines, TGF-1, and anti-OVA IgE in the coadministration group (Additional file 1: Fig. 4), which showed that CpG-ODNs potentiated the effects of corticosteroids.

**Fig. 2:**
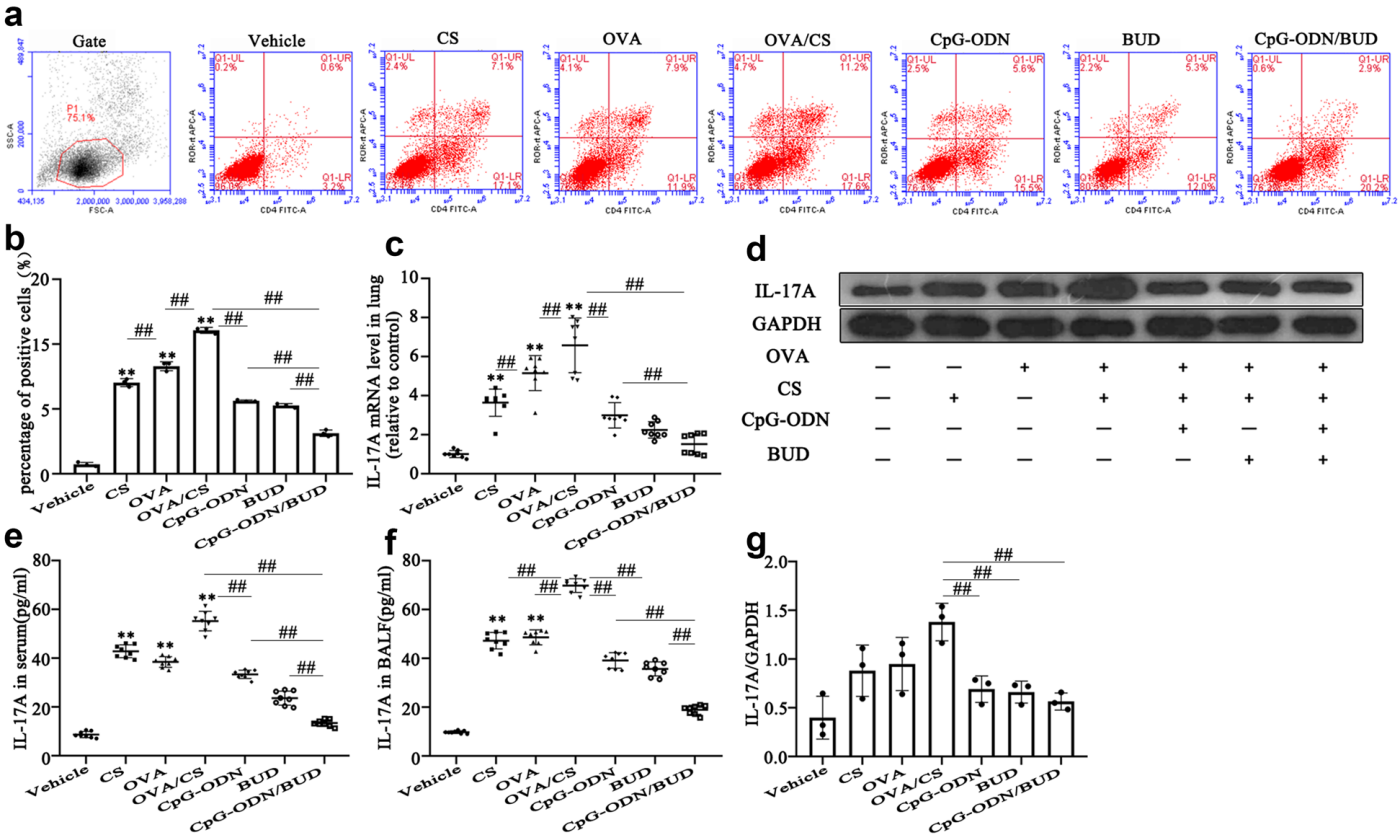
CpG-ODN and BUD synergistically alter Th17 responses in the lower airway. RORt^+^CD4^+^ cells representing Th17 cells were assessed flow-cytometrically (**a**) and positive cells were quantitated (**b**). Th17-related cytokine amounts in serum (**e**) and BALF (**f**) were determinedby ELISA, and relative mRNA expression was measured by qRT-PCR in lung tissues from mice (**c**). Immunoblot was carried out for assessing the protein amounts of Th17-associated cytokinesinlung tissues, with GAPDH as a loading control (**d**); HDAC2/GAPDH ratios were assessed (**g**). Statistical significance denoted:^*, #^*p*<0.05, ^**,##^*p*<0.01. *versus the vehicle control group; # versus the indicated group

Th17 cells exert their effects by producing multiple inflammatory cytokines such as IL-17 A, which is known to enhance the chemotaxis of neutrophils toward bronchial epithelial cells and airway smooth muscle cells [29]. Increasing evidence has claimed that Th17-associated neutrophilic airway inflammation in the mouse is GCs insensitive [30]. As expected, Th17 cells in CS-exposure asthmatic mice were markedly elevated compared with those in the vehicle control group, according to the flow cytometry data (Fig. 2a, b). Moreover, significantly elevated protein and mRNA levels of IL-17 A in serum, lung, and BALF were found in the CS, OVA, and OVA/CS groups compared with the vehicle control group (Fig. 2cg). Both CpG-ODNs and BUD decreased the percentage of Th17-positive cells, and IL-17 mRNA and protein levels compared with those in untreated CS-induced asthmatic mice (Fig. 2ag). Meanwhile, combined treatment with CpG-ODNs and BUD markedly reduced Th17 cells, IL-17 mRNA and protein levels compared with those in the monotherapy groups (Fig. 2ag).

Taken together, these data indicated that CS-exposure associated asthma induced a Th17/Th2-type response, and CpG-ODNs and BUD synergistically decreased the exacerbated levels of Th17- and Th2-associated cytokine, and enhanced the biosynthesis of the Th1-associated cytokine IFN-.

### **HDAC2 activity and expression restoration upon treatment with CpG-ODNs and BUD in CS-exposure asthmatic mice**

**Fig. 3:**
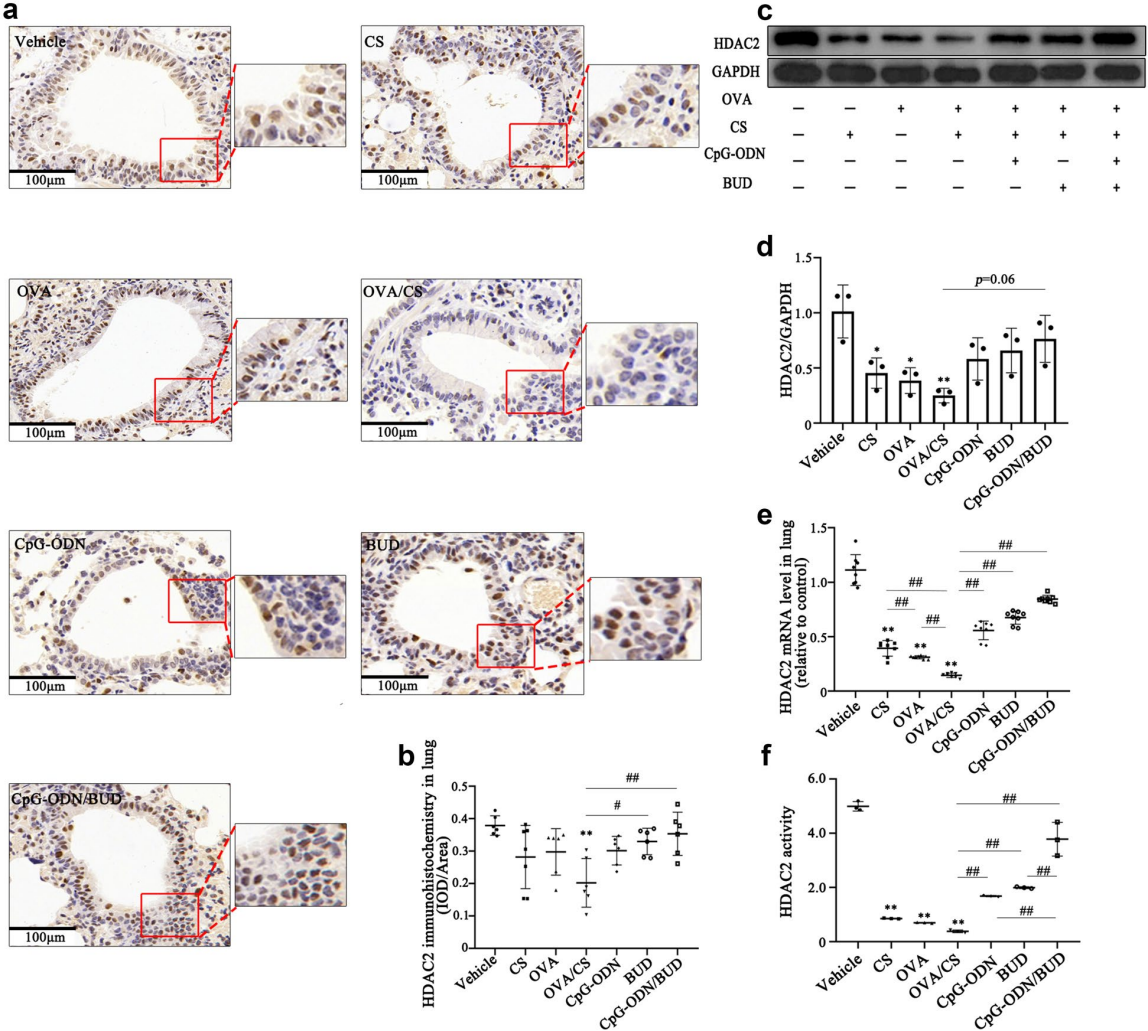
CpG-ODN and BUD synergistically affect HDAC2 activity and expression in chronic CS-exposed asthmatic mice. Representative photomicrographs of immunohistochemical staining of lung sections for HDAC2 in various animal groups (200) (**a**). HDAC2-positivity rate was determined as HDAC2-positive area/total bronchiole area (**b**). Immunoblot was carried out for assessing HDAC2 protein amounts in lung tissue specimens, with GAPDH as a loading control; HDAC2/GAPDH ratios were assessed (**c**, **d**). The mRNA expression levels of HDAC2 were evaluated by qRT-PCR in lung tissue samples from mice (**e**). HDAC2 activity was determined with the colorimetric EpiQuik HDAC2 Activity Assay Kit (**f**) .Statistical significance denoted:^*, #^*p*<0.05, ^**,##^*p*<0.01. ^*^ versus the vehicle control group; ^#^ versus the indicated group

CS reduces responsiveness to steroids by modifying histone acetyltransferase, which is an essential epigenetic enzyme that mediates the anti-inflammatory effects of steroids [31, 32]. Furthermore, HDAC2 activity and levels are substantially decreased by oxidative/nitrative stress, causing insensitivity to the anti-inflammatory effects of GCs [33]. In this study, we assessed the levels of secreted HDAC2 in lung tissue samples by immunohistochemistry, ELISA and Western blotting. As shown in Fig. 3, OVA challenge and CS exposure both markedly decreased HDAC2 mRNA and protein levels (Fig. 3ae). We also investigated the effects of CpG-ODNs and BUD on CS-induced changes in HDAC2 mRNA and protein expression levels to verify whether CpG-ODNs affect HDAC2 expression. Interestingly, it was found that after treatment with CpG-ODNs or BUD only, the effect on HDAC2 gene expression levels were with the opposite of those in the untreated group, and this effect was enhanced after coadministration of CpG-ODN and BUD (Fig. 3ac, e), however, a nonsignificant increase in HDAC2 protein expression levels was observed in mice that were administered CpG-ODN plus BUD (*p*=0.06, Fig. 3d).

Moreover, based on studies reporting that patients with severe asthma have diminished GCs sensitivity in peripheral blood monocytes (PBMCs) in comparison with patients with nonsevere asthma, in association with decreased HDAC2 activity that parallels the impairment in GCs sensitivity [34], we analyzed HDAC2 activity with an HDAC2 activity assay kit. As expected, similar to HDAC2 expression, HDAC2 activity in OVA+CS challenged mice was obviously suppressed and markedly recovered after the administration of CpG-ODN or BUD, with significant differences between the OVA/CS and CpG-ODN/BUD groups, reflecting the changes in HDAC2 protein expression (Fig. 3f) .

These data suggested that the expression and activity of HDAC2 was impaired in chronic asthmatic murine models. Meanwhile, CpG-ODNs restored responsiveness to GCs therapy by restoring HDAC2 expression and enhancing HDAC2 activity. When combined with BUD, CpG-ODNs restored HDAC2 activity and expression more substantially than either CpG-ODNs or BUD alone.

### Decreased RORt expression and Th17 responses in response to CS and OVA challenge after CpG-ODNs and BUD treatment

**Fig. 4:**
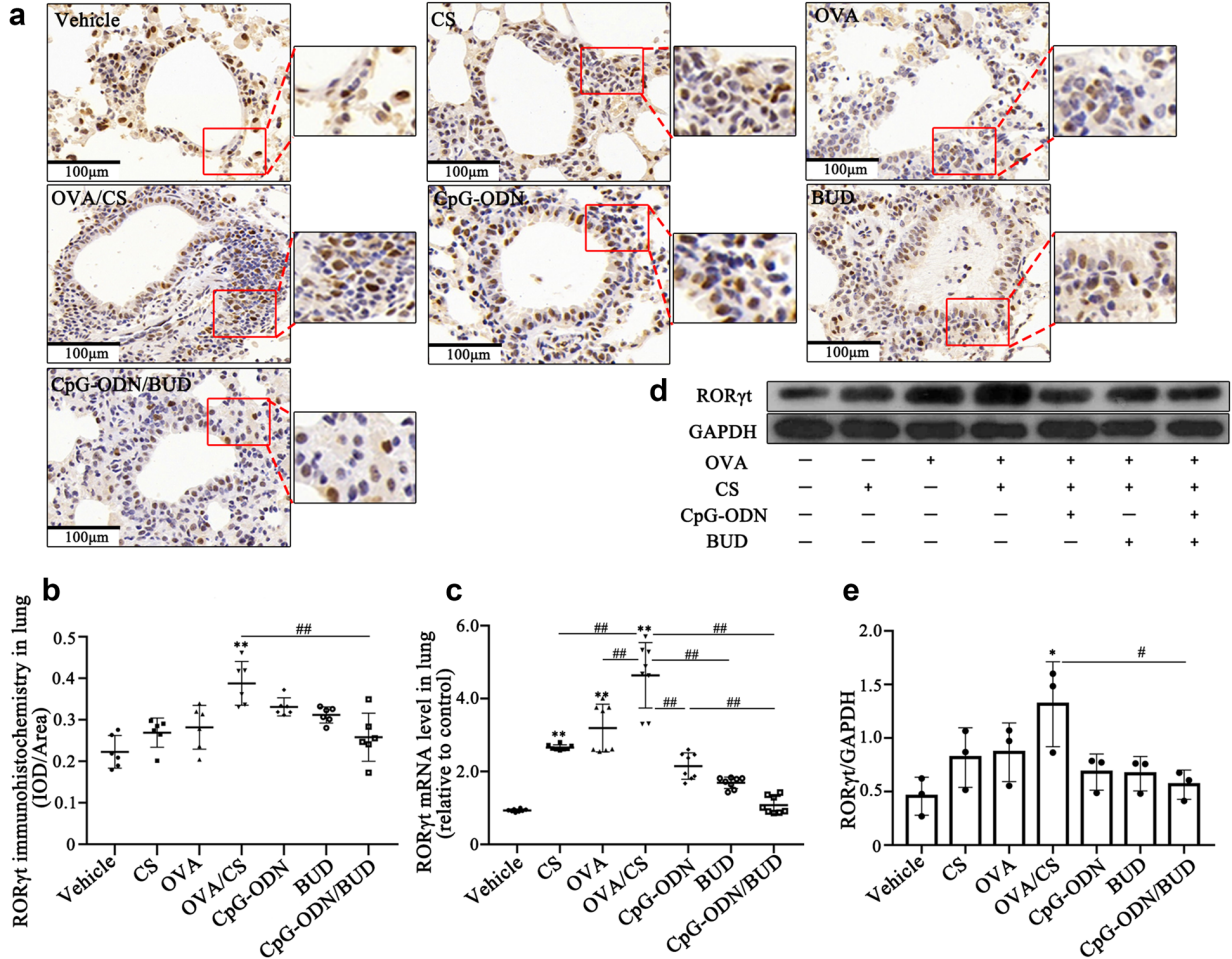
CpG-ODN and BUD synergistically affect RORt expression in CS-exposed asthmatic mice. Representative photomicrographs of immunohistochemical staining of lung sections for RORt from various animal groups (200) (**a**). RORt-positivity rate was determined as RORt-positive area/total bronchiole area (**b**). The mRNA expression levels of RORt were determined by qRT-PCR in lung tissue samples from mice (**c**). Immunoblot was carried out for assessing RORt protein levels in lung tissue samples, with GAPDH as a loading control; RORt/GAPDH ratios were determined (**d**, **e**). Statistical significance denoted:^*, #^*p*<0.05, ^**,##^*p*<0.01. ^*^versus the vehicle control group; ^#^ versus the indicated group

HDAC2 is important in Th-17 cell differentiation from naive CD4^+^ T cells, and RORt involvement in this process has attracted increasing attention [35, 36]. The catalytic activity of HDAC2 is important in inhibiting RORt transcriptional activity, and SUMOylated RORt recruits HDAC2 to the IL-17 promoter for gene downregulation [37]. To explore the mechanism by which CpG-ODNs treatment regulates the cytokine IL-17 A due to HDAC2 upregulation, we next examined the levels of RORt, which is an important biomarker of the HDAC2-mediated Th17 response in CS-induced asthma, by immunohistochemistry, ELISA and Western blotting. The results showed a distinct increasing trend in RORt mRNA and protein expression levels in CS-exposed asthmatic mice in comparison with vehicle control mice (Fig. 4). Meanwhile, upon combined administration of CpG-ODNs and BUD, the animals showed significantly decreased RORt mRNA and protein levels (Fig. 4), indicating that CpG-ODNs combined with BUD suppressed RORt to a certain extent, thereby inhibiting IL-17 A expression in Th17 cells.

### CpG-ODNs and BUD synergistically regulate the interplay between HDAC2, RORt and IL-17 A, orchestrating inflammatory reactions in HBE cells

Airway epithelial cells play a critical role on in defense against allergens, viruses, and environmental pollutants, which are involved in asthma pathogenesis. Moreover, IL-17 A is found in airway epithelial cells [38]. To further confirm whether CpG-ODNs inhibit the RORt-mediated Th17 response via HDAC2, we next examined *in vitro* cultures of HBE cells exposed to OVA and/or CSE, that were administered CpG-ODNs and/or BUD. We performed ELISA, qRT-PCR, Western blotting, immunofluorescence analysis and flow cytometry to assess the levels of cytokines, HDAC2 and RORt in all groups.

**Fig. 5:**
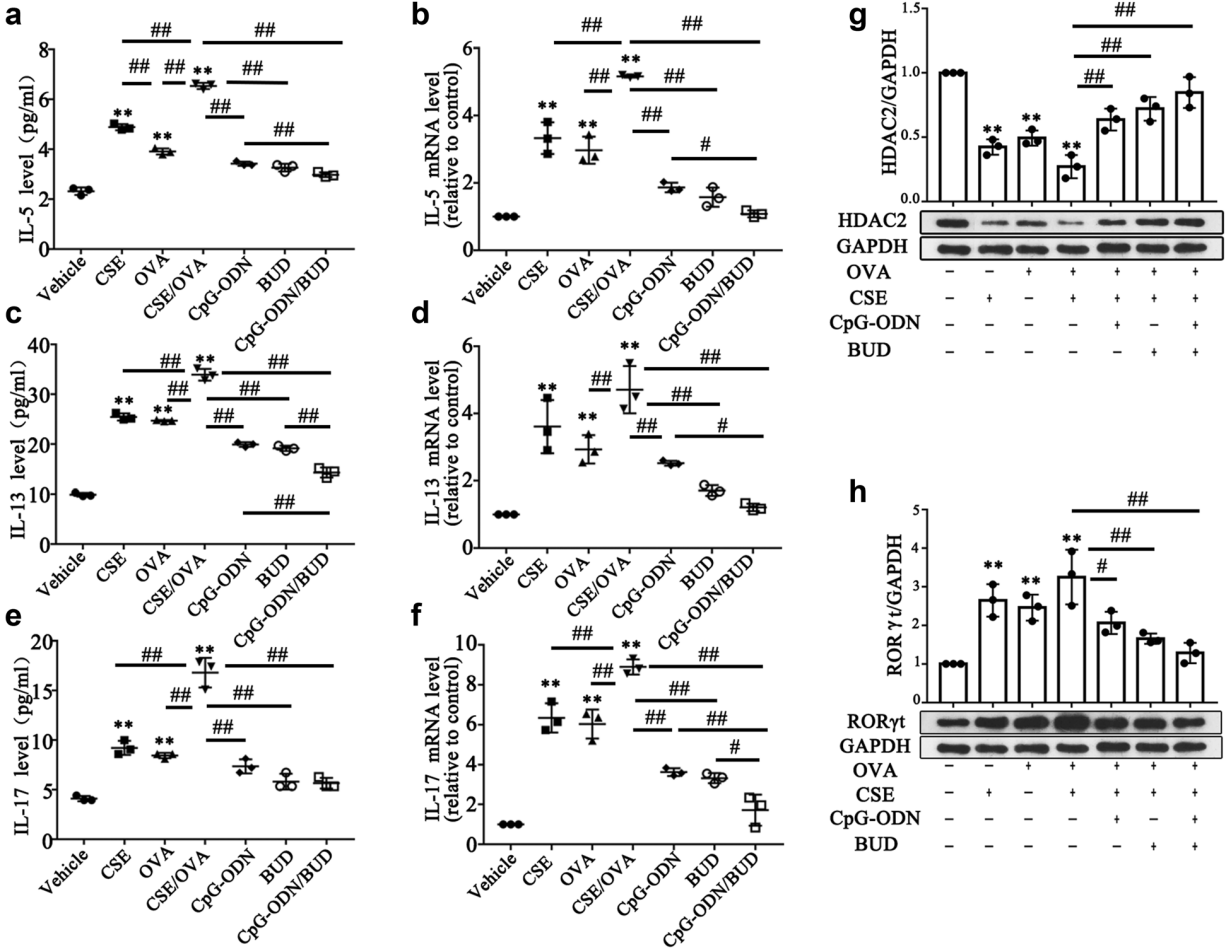
CpG-ODN and BUD synergistically regulate the interplay of HDAC2, RORt and IL-17 A, orchestrating the inflammatory response in HBE cells. Th2-associated cytokines (**a**, **c**) examined by ELISA, and relative mRNA levels of Th2 cytokines in lung tissue specimens from mice (**b**, **d**), determined by qRT-PCR. Th17-associated cytokines (**e**) assessed by ELISA, and relative mRNA levels of Th2 cytokines in lung tissue samples from mice (**f**), evaluated by qRT-PCR.Immunoblot was carried out for assessing the protein amounts of HDAC2 (**g**) and RORt in HBE cells, with GAPDH as a loading control; HDAC2/GAPDH (**g**) and RORt/GAPDH (**h**) ratios were determined. Statistical significance denoted:^*, #^*p*<0.05, ^**,##^*p*<0.01. ^*^ versus the vehicle control group; ^#^ versus the indicated group

Consistent with the animal data, CSE-exposed or OVA-challenged HBE cells had elevated IL-5, IL-13 (Th2 cytokines) and IL-17 A (Th17 cytokine) levels compared with those in the vehicle control group. These cytokines were markedly increased in HBE cells after co-exposure to CSE and OVA (all *p<*0.01, Fig. 5af). Moreover, HBE cells to OVA and CSE alone or in combination significantly reduced HDAC2 levels and markedly increased RORt and IL-17 A gene and protein levels, suggesting specific associations of HDAC2 and RORt with the IL-17 promoter in HBE cells (all *p<*0.05, Figs. 5gh and 6). Since HDAC2 is the main HDAC that contributes to the effects of GCs, whether CpG-ODNs influence the interplay between HDAC2, RORt and IL-17 A in HBE cells was examined. OVA- and CSE-exposed HBE cells were treated with CpG-ODNs and BUD. Interestingly, after administration of CpG-ODNs or BUD, HDAC2 protein levels showed an increasing trend. Notably, the increasing trend in HDAC2 expression changes was more pronounced after combined treatment with CpG-ODNs and BUD. However, contrary to the HDAC2 results, RORt and IL-17 A levels were decreased in mice that were administered CpG-ODNs and were notably reduced after the co-administration of BUD and CpG-ODNs (all *p<*0.05, Figs. 5gh and 6).

**Fig. 6:**
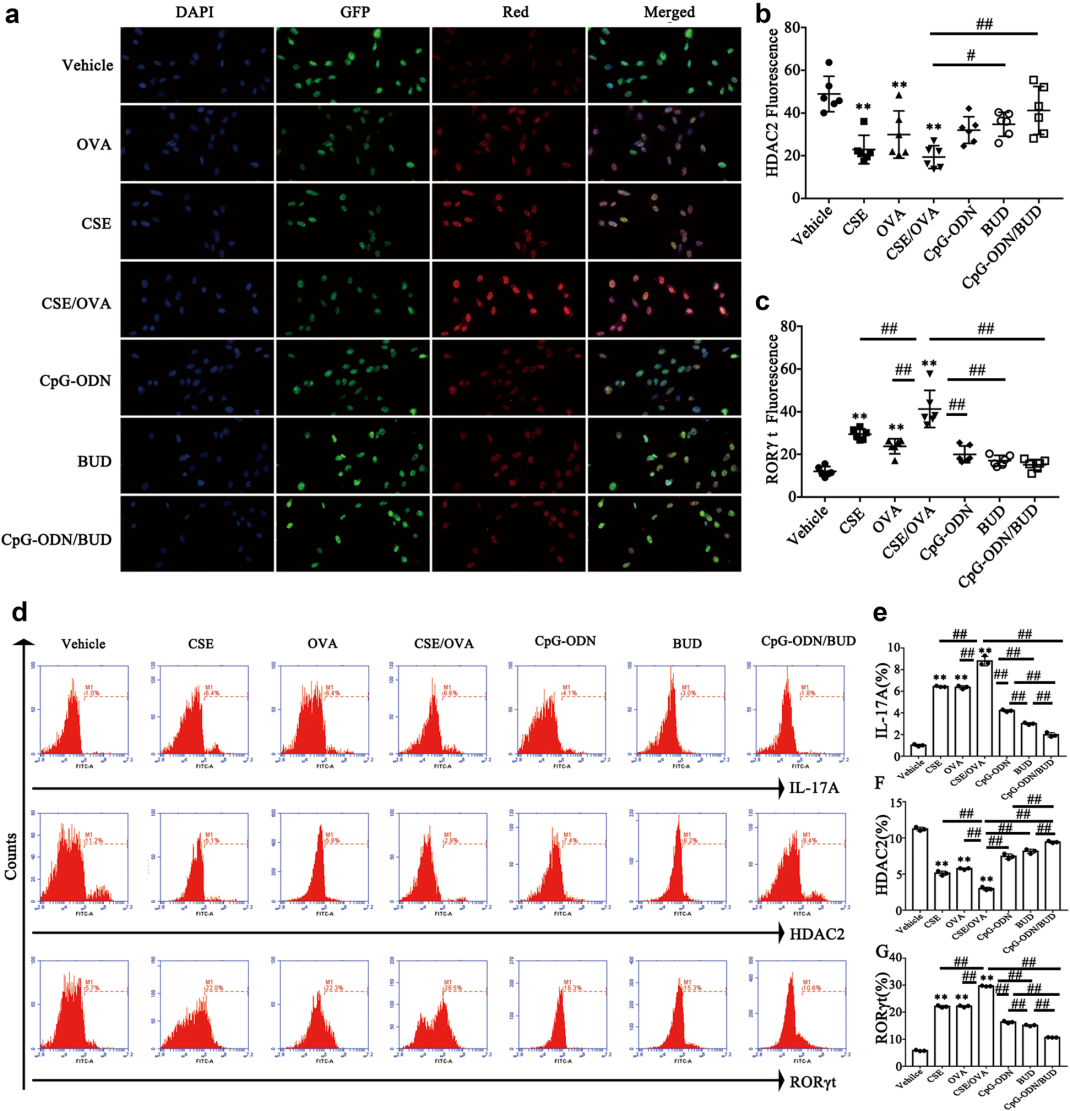
CpG-ODN and BUD synergistically affect HDAC2, RORt and IL-17 A in HBE cells. Representative micrographs of immunofluorescent staining of HBE cells for detecting tubulin GFP(green; HDAC2-positive) and H2BmCherry(Red; RORt-positive) cells (200) (**a**). HDAC2-positivity rate was determined as tubulin GFP(green)-positive area/total area (**b**). RORt-positivity rate was evaluated as H2BmCherry(Red)-positive area/total area (**c**). Representative images depicting IL-17 A^+^, HDAC2^+^, and RORt^+^ cells in HBE cell populations (**d**). The ratios of specific protein-positive HBE cells to total HBE cells (**e**-**g**) were assessed by flow cytometry. Statistical significance denoted:^*, #^*p*<0.05, ^**,##^*p*<0.01. ^*^ versus the vehicle control group; ^#^ versus the indicated group

Collectively, these data provide convincing evidence of an interplay between HDAC2 and RORt in OVA-induced and CSE-exposed airway epithelial cells, which substantially affects allergic airway inflammation. Moreover, CpG-ODNs could partly affect this interplay, by simultaneously improving HDAC2 expression and inhibiting RORt expression.

## Discussion

Our previous study and others revealed that CS-exposed asthma exhibits elevated inflammatory cell infiltration [10, 31], mucus production, airway remodeling, and Th2/Th17 polarization, which was further confirmed in the current study (Figs. 1 , 2 and Additional file 1: Figs. S3 and S4). As shown in Fig. 1, immunohistochemical analysis of Gr-1 (neutrophil-specific marker) and ECP (eosinophil-specific marker) confirmed a substantially increase in neutrophil and eosinophil influx into the lung, which suggested that CS and OVA induced the infiltration of inflammatory cells, including eosinophils and neutrophils, into pulmonary tissues. Neutrophilic inflammation is driven by IL-17, TNF-, and IL-8 [39]. This study showed that IL-17 and neutrophil-associated chemokines, including IL-8 and TNF-, were significantly elevated in the OVA/CS group, which may account for the enhanced infiltration of neutrophils into pulmonary tissue. In addition, eotaxin 1 was increased in the BALF in the CS/OVA group (Fig. 1). Eotaxin 1 promotes the recruitment of eosinophils and other immune cells, such as neutrophils [40]. Increasing evidence suggests that decreased sensitivity to GCs is associated with neutrophilic airway inflammation, and steroid-insensitive asthma is characterized by Th17 cytokines with neutrophilic inflammation [41, 42]. In this study, BUD alone markedly decreased Th2/Th17 cytokines in BALF and reduced immune cells in pulmonary tissue (Figs. 1 , 2 and Additional file 1: Figs. S3 and S4), although to a lesser extent than that observed in our previous study [10]. These findings indicate that the current model is not GCs resistant but rather GCs insensitive in the context of lung cell inflammation. According to these findings, mice co-exposed to CS and OVA showed exaggerated reactions to allergen inhalation, triggering inflammation that simultaneously involved eosinophils and neutrophils, elevated type 17-associated immune responses, and relative insensitivity to GCs.

Our previous study demonstrated that CpG-ODNs alleviate mixed airway neutrophil and eosinophil inflammation in CS exposure OVA-induced asthma [10]. Several reports have revealed decreased HDAC2 activity in smokers and the sputum cells of patients with respiratory diseases, as well as in CS-exposed asthma mice [19, 25, 32, 43], indicating that insufficient transcriptional corepressor levels and activity could be critical for asthma pathogenesis [44]. Emerging evidence has suggested that theophylline could downregulate the inflammatory response, locally and systemically, by increasing HDAC2 activity in patients with asthma [15, 45]. Since CpG-ODNs reduce the inflammatory response, we examined whether CpG-ODNs modulate HDAC2 activity and expression, thereby enhancing the response to GCs in CS-exposure asthma mice. In the current CS exposure OVA-induced model, both HDAC2 mRNA and protein levels or activity were markedly suppressed, and as expected, these factors were increased after CpG-ODNs administration. Furthermore, the combination of CpG-ODNs and BUD more substantially restored HDAC2 activity and expression, which may account for the amelioration of corticosteroid insensitivity. Interestingly, consistent with another study [46], BUD robustly suppressed mediator release compared to the enhancement in HDAC2 activity [15, 47]. CpG-ODNs may act in an indirect manner with corticosteroids to increase its effect on HDAC2 activity, suggesting that CpG-ODNs exert corticosteroid-sparing effects.

There is increasing evidence that Th17 lymphocytes play a critical role in inducing neutrophilic airway inflammation. Th17 cells produce various inflammatory cytokines, including IL-17 A, which regulates cellular immunity by upregulating downstream proinflammatory molecules in epithelial and mesenchymal cells, thereby mediating neutrophil infiltration and activation, and promoting neutrophil accumulation in pulmonary tissues. Interestingly, in the present study, opposite trends in between HDAC2 and IL-17 A were found, with HDAC2 downregulated while IL-17 A was upregulated in CS-exposure asthmatic mice. Several studies have shown that the striking interaction between HDAC2 and IL-17 A forms a vicious cycle, leading to the exacerbation of asthma [43]. According to a study by Lai et al. [43], HDAC2 impairment upregulates IL-17 A, and IL-17 A deficiency ameliorates the reduction in HDAC2, suggesting that HDAC2 is a mediator that affects the secretion of IL-17 A, thereby causing a Th17-polarized response. In the current study, CS-exposed mice that were administered CpG-ODNs had elevated HDAC2 expression and attenuated IL-17 A production. Collectively, CpG-ODNs is likely involved in the interaction between HDAC2 and IL-17 A. Therefore, the current results indicate that CpG-ODNs may affect IL-17 A secretion by modulating HDAC2 activity and expression, thereby inhibiting the Th17 response.

On the other hand, RORt is an important transcription factor that regulates IL-17 A [48]. Singh et al. described a detailed mechanism by which the SUMOylation of RORt promotes HDAC2 interactions with the IL-17 promoter and suppresses IL-17 A transcription [36]. Furthermore, a study demonstrated RORt acetylation in Th17 cells and this effect was significantly enhanced by HDAC2 inhibitors [49], which was consistent with a recent study that revealed elevated RORt acetylation in cultured CSE-induced HBE cells with HDAC2 silencing [25]. Based on these findings, HDAC2 may inhibit RORt-mediated IL-17 A production, thereby attenuating the Th17 response. Consistent with our hypothesis, the results indicated a negative correlation between HDAC2 and RORt expression in CS-exposure asthmatic mice. Therefore, we hypothesized that CpG-ODNs might mainly accelerate the binding capacity of RORt to IL-17 A via HDAC2, to inhibit IL-17 A expression.

Bronchial epithelial cells are increasingly thought to contribute to innate immunity. We hypothesized that altered sensitivity to GCs in airway epithelial cells is substantially involved in GCs insensitivity in inflammatory responses [50]. Consistent with a recent study [9], we found that CSE exposure markedly downregulated HDAC2 expression in HBE cells, and costimulation with OVA and CSE in asthmatic conditions further deceased HDAC2 expression (Figs. 5 and 6). Contrary to the change in HDAC2 expression, elevated RORt expression was observed in HBE cells cultured with CSE, with markedly increased expression in HBE cells that were administered both CSE and OVA (Figs. 5 and 6). These results indicated an interplay between HDAC2 and RORt in OVA- and CSE-induced airway epithelial cells, suggesting an essential function of these factors in Th17 inflammation. Next, whether CpG-ODNs regulate HDAC2 and RORt in airway epithelial cells to change the sensitivity of these cells to GCs was investigated. We treated HBE cells induced with OVA and CSE with CpG-ODNs and BUD and found that CpG-ODNs regulated the interplay between HDAC2 and RORt, synergistically with BUD to some extent, which was consistent with the effects in the CS-exposure asthmatic murine model (Figs. 5 and 6). Of note, due to relatively expensive, low storage temperature, and possible biosafety consideration, we used complete culture medium supplemented with 10% FBS, instead of the growth medium recommended by the ATCC, to maintain HBE cells *in vitro*, which may be a potential limitation of this study. However, HBE cells showed uniform and stable growth in a continuous and consistent manner (Additional file 1: Fig. S2), demonstrating that HBE cells were cultured successfully. It is known that IL-17 A modulates the protective effects of HDAC2 on airway inflammation in asthma and that HDAC2 activation and/or IL-17 A downregulation can prevent allergic airway inflammation [43]. Moreover, RORt transcriptionally upregulates IL-17 A, indicating that CpG-ODNs may suppress RORt-mediated IL-17 A expression via HDAC2 *in vitro*, which is consistent with the above evidence.

## Conclusions

Overall, CpG-ODNs and BUD synergistically improved adverse CS-exposure asthma outcomes and inhibited, at least in part, RORt-mediated Th17 response by restoring HDAC2 expression and activity, consequently ameliorating GCs insensitivity. These data suggest that CpG-ODNs may have therapeutic value in reviving steroid effects in CS-exposure asthma, providing new insights into the mechanism by which CpG-ODNs improve sensitivity to steroids.

## Supplementary Information

**Additional file 1: Table S1.** Sequence of primers for qRT-PCR.** Fig. S1.** Experimental protocol for the study.** Fig. S2.** HBE Cell viability varies with increasing concentration and time.** Fig. S3.** CpG-ODNs and BUD inhibit mucus secretion and airway structural remodeling in mice induced by OVA-challenge and CS-exposure.** Fig. S4.** CpG-ODNs and BUD synergistically alter Th1/Th2 type responses in the lower airway.

## Data Availability

The datasets used and/or analyzed during the current study are available from the corresponding author on reasonable request.
