# Supplementary material for: CpG oligodeoxynucleotides attenuate RORt-mediated Th17 response by restoring histone deacetylase-2 in cigarette smoke-exposure asthma
Source: Cell Biosci. 2021 May 20;11:92. doi: 10.1186/s13578-021-00607-3 (PMC8139164; doi:10.1186/s13578-021-00607-3)
Supplement: Supplementary file 1 — Additional file 1: Table S1. Sequence of primers for qRT-PCR. Fig. S1. Experimental protocol for the study. Fig. S2. HBE Cell viability varies with increasing concentration and time. Fig. S3. CpG-ODNs and BUD inhibit mucus secretion and airway structural remodeling in mice induced by OVA-challenge and CS-exposure. Fig. S4. CpG-ODNs and BUD synergistically alter Th1/Th2 type responses in the lower airway. [file 13578_2021_607_MOESM1_ESM.doc]

**Supplementary materials and methods**

**Mice and animal experiments**

The animals were sensitized with 50 μg of OVA (grade V; Sigma-Aldrich, USA) or NS emulsified with 2 mg of aluminum hydroxide (YuanMu, China) in 200 μl of NS at days 0, 7, 14, and 21. From days 28 to 70, we placed the mice in the CS, OVA/CS, CpG-ODN, BUD and CpG-ODN/BUD groups in a self-made box (45× 55× 40 cm^3^) in which the mice were subjected to CS via whole-body ventilation of commercial cigarettes (per cigarette: tar oil, 10 mg; nicotine, 1.0 mg; carbon monoxide, 13 mg) produced by China Tobacco Hunan Industrial Corporation (China) from which the filters were removed. Two 30-min cigarette exposures were performed daily at 12-h intervals for 5 days/week, while mice in the vehicle control and OVA groups underwent exposure to ambient air.

In addition, after 30 min air/CS exposure, the animals were administered OVA (3%) or NS by aerosol for 30 min. In the CpG-ODN group, 30 min post-challenge, the animals received intranasal CpG-ODNs (40 μg/nostril, Sangon Biotec., China) according to a previous report, while in the BUD group, animals were intranasally administered with BUD (10 μg/nostril in 20 μL; Budesonide Inhalation Suspension, AstraZeneca, UK) 30 min after OVA challenge. The animals in the CpG-ODN/BUD group received intranasal CpG-ODN and BUD in succession after 10 min. Mice underwent euthanasia at 24 h after allergen challenge. The doses of CpG-ODN and BUD were determined in pilot studies [1-4] and other reports [5-8]. Aschematic diagram of the CS-exposure asthmatic murine model and treatments is shown in Supplementary e-Fig. 1.

**Bronchoalveolar lavage fluid (BALF) collection and quantification of cytokines**

Cell counts in BALF specimens collected from mice were obtained as previously described [2, 4, 9]. BALF levels of interleukin 8 (IL-8), IL-5, IL-13, interferon-gamma (IFN-γ), IL-17A, transforming growth factor-beta (TGF-β1), tumor necrosis factor-α (TNF-α) and eotaxin1 were assessed with specific enzyme-linked immunosorbent assay (ELISA) kits (Bioss Inc., China) according to the manufacturer’s protocols.

**Histological examination and immunohistochemistry**

Lung specimens from all groups were extracted and sectioned. Hematoxylin-eosin (H&E) staining was carried out to evaluate the inflammatory status. Mucus production by goblet cells was assessed by periodic acid-Schiff (PAS) staining sections, and the results were semiquantitated (score:0-5) as previous described [10].The area of peribronchial Masson’s trichrome staining (blue) was visualized and quantified by the software as a percentage of the total band area. The slides were incubated with either a rabbit polyclonal antibodies against Gr-1, ECP, HDAC2 (Invitrogen, USA) or RORγt (Invitrogen, USA). Quantitative measurements of Gr-1-, ECP-, HDAC2- or RORγt- positive cells in the lung tissue were performed according to previously described methods[11, 12]. Briefly, the numbers of positive and negative cells were counted in each specimen. Lung cells that were positive for the Gr-1, ECP, HDAC2, and RORγt antibody staining are expressed as a percentage of the total cells. Collagen-positive areas were quantified by Image-Pro 6.1 software (Media Cybernetics).

**Western blot analysis**

Proteins in lung tissue lysates were isolated by TissueLyser II (QUIAGEN, Germany) and immunoblotted using antibodies against the following proteins: IL-17A, HDAC2, RORγt and β-actin (Invitrogen, USA).

**Serum IL-17A and OVA-specific IgE level evaluation**

Serum IL-17A and OVA-specific IgE levels were assessed with ELISA kits (Bioss Inc., China) as directed by the manufacturer.

**Quantitative reverse transcription polymerase chain reaction (qRT-PCR)**

Total RNA from left lung specimens was obtained with TRIzol reagent (Invitrogen, USA). Real-time PCR was performed for 50 cycles on a Step One Real-time PCR System (Applied Biosystems). The sequences of the primers are listed in Supplementary Table 1.

**Lung function measurement**

AHR was determined 24 h after the final OVA administration and/or CS exposure by whole-body plethysmography (Buxco Electronics, USA) using a previously described method [13]. AHR data expressed by enhanced pause (Penh) are represented as airway resistance at 100 mg/mL methacholine.

**Flow cytometry**

Flow cytometry was carried out essentially as previously described [1]. Mouse-specific monoclonal antibodies (Invitrogen,USA) included IL-17A-labeled CD4 (Invitrogen, USA), HDAC2-labeled CD4 (Invitrogen, USA) and RORγt-labeled CD4 (Invitrogen, USA). In addition, flow cytometry was performed with a Fortessa (BD Biosciences), and the data were analyzed with FlowJo (TreeStar).

Supplementary Table1.Sequence of primers for qRT-PCR

| Gene | **5'to 3' Sequence** | Size |
| --- | --- | --- |
| IL-17A | TTTAACTCCCTTGGCGCAAAA | 253 bp |
|  | CTTTCCCTCCGCATTGACAC |  |
| IL-13 | ACCCAACAACCACCTATGCT | 150 bp |
|  | TGCACTCATTGGTGGAGGTA |  |
| IL-8 | CTTGTCATTGCCAGCTGTGT | 136 bp |
|  | TGACTGTGGAGTTTTGGCTG |  |
| HDAC2 | TGCTTGCCATCCTCGAATTA | 149 bp |
|  | AGGAAAGTATTCCCCATATT |  |
| RORγt | GCAGGAGCAATGGAAGTCG | 162 bp |
|  | CGCTGAGGAAGTGGGAAAA |  |
| GAPDH | TGGCCGTGGGGCTGCCCAG | 107 bp |
|  | GGAAGGCCATGCCAGTGAGC |  |

**
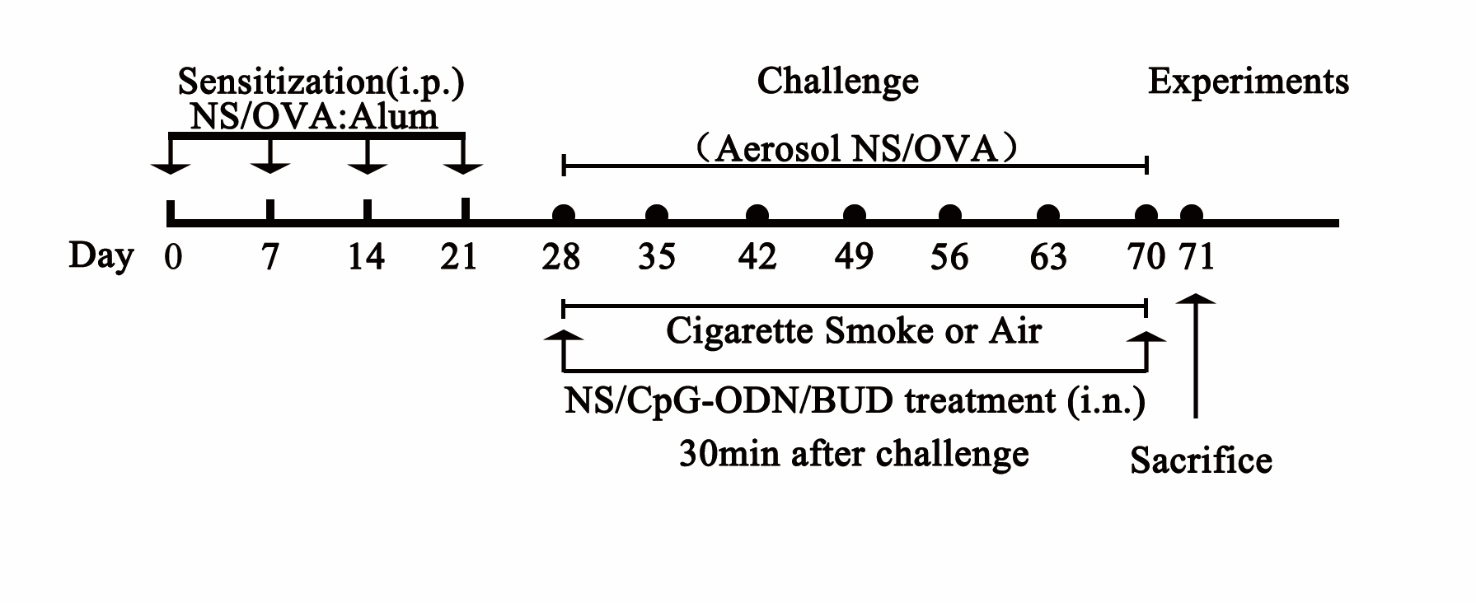
**

**e-Fig.1.Experimental protocol for the study.** Mice were sensitized to ovalbumin (OVA) or NS by four intraperitoneal (i.p.) injections on days 0, 7, 14 and 21 with OVA (50 μg) or NS absorbed to alum (20 mg). This sensitization was followed by five days per week of OVA or NS aerosol challenge for 6 consecutive weeks as indicated. Mice were chronically exposed to cigarette smoke or air twice per day, five-weekly for 6 weeks. Therapeutic treatment with CpG-ODNs and BUD (both intranasal) was started 30 min after aerosol challenge. Twenty-four hours after the final allergen challenge, the mice were sacrificed by cervical dislocation, and experiments were performed.

**
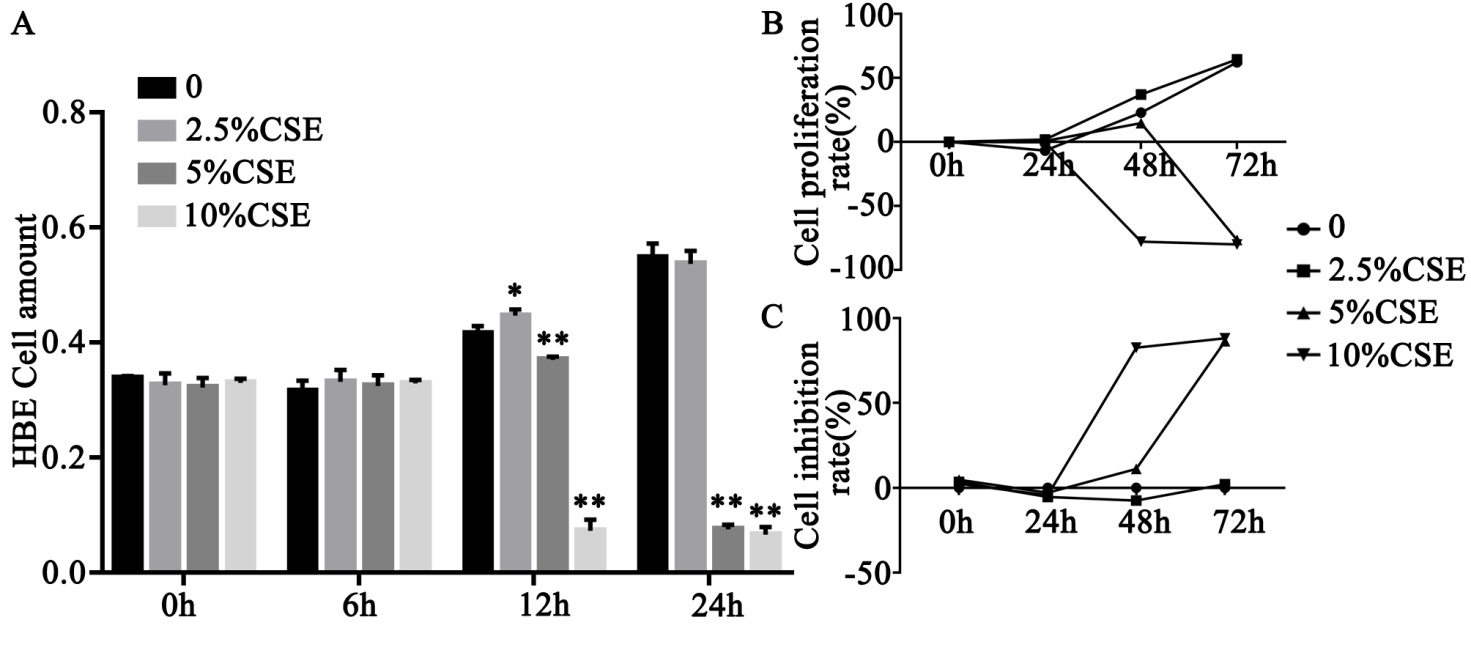
**

**e-Fig.2. HBE Cell viability varies with increasing concentration and time.** HBE cells were stimulated with increasing concentrations of CSE for increasing times (A). The proliferation (B) and inhibition rate (C) of HBE cells are shown at different concentrations over time.

**e-Fig.3.CpG-ODNs and BUD inhibit mucus secretion and airway structural remodeling in mice induced by OVA-challenge and CS-exposure.** Representative histological images of H&E-stained lung tissue at a magnification of 200×(A). Representative photomicrographs of lung sections stained with periodic acid-Schiff (PAS) for assessing mucus deposition (B). Original magnification, 200×. Representative photomicrographs of Masson’s trichrome-stained lung sections in the indicated groups(C). Original magnification, 200×. Semiquantitative PAS scores of lung samples were obtained as described in the main text (D).The collagen-positive area was calculated as the collagen-positive area/total bronchiole area in each group (E).Airway resistance to methacholine was measured at 24 h after the final OVA challenge (F). Statistical significance denoted:^*, #^*p*<0.05, ^**,##^*p*<0.01,where * represents a difference between any sample relative to the vehicle control group; # represents a difference between the indicated groups.

**
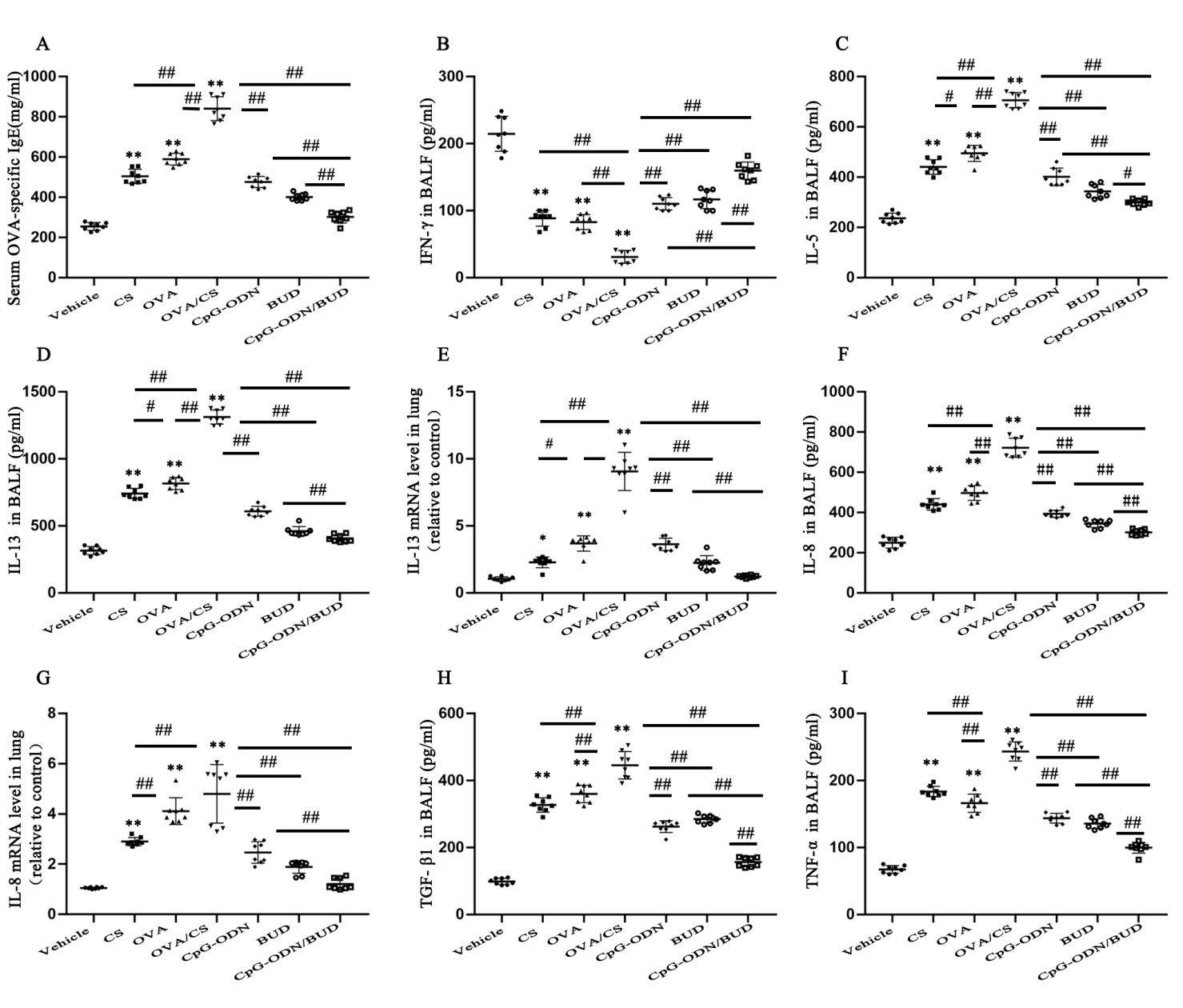
e-Fig.4.CpG-ODN and BUD synergistically alter Th1/Th2 type responses in the lower airway**. ELISA was used for the detection of OVA specific IgE in serum (A). Th1-associated cytokine was measured by ELISA (B). The amounts of Th2-associated cytokines in the BALF at protein levels (C,D) and in the lung tissues at mRNA level (E) were measured by ELISA and qRT-PCR, respectively. IL-8 level in the BALF (F), as measured by ELISA, and relative mRNA expression levels of IL-8 in lung tissues from mice (G), as assessed by qRT-PCR. TGF-β1 (H) and TNF-α (I) levels in the BALF were measured by ELISA. Data are mean ± standard deviation (SD). Statistical significance denoted:^*, #^*p*<0.05, ^**,##^*p*<0.01,where * represents a difference between any sample relative to the normal saline (NS) control group; # represents a difference between the indicated groups.

**References**

1. Li HT, Lin YS, Ye QM, Yang XN, Zou XL, Yang HL, Zhang TT. Airway inflammation and remodeling of cigarette smoking exposure ovalbumin-induced asthma is alleviated by CpG oligodeoxynucleotides via affecting dendritic cell-mediated Th17 polarization. Int Immunopharmacol.2020, 82:106361.
2. Li HT, Chen ZG, Liu H, Ye J, Zou XL, Wang YH, Yang HL, Meng P, Zhang TT. Treatment of allergic rhinitis with CpG oligodeoxynucleotides alleviates the lower airway outcomes of combined allergic rhinitis and asthma syndrome via a mechanism that possibly involves in TSLP. Exp Lung Res. 2016;42:322-33.
3. Li HT, Chen ZG, Lin YS, Liu H, Ye J, Zou XL, Wang YH, Yang HL, Zhang TT. CpG-ODNs and budesonide act synergistically to improve allergic responses in combined allergic rhinitis and asthma syndrome induced by chronic exposure to ovalbumin by modulating the TSLP-DC-OX40L axis. Inflammation. 2018;41:1304-20.
4. Li HT, Zhang TT, Chen ZG,Ye J, Liu H, Zou XL, Wang YH, Yang HL.Intranasaladministration of CpG oligodeoxynucleotides reduces lower airway inflammation in amurinemodel of combined allergic rhinitis and asthma syndrome. Int Immunopharmacol.2015;28 (1): 390-8.
5. Pesce I, Monaci E, Muzzi A, Tritto E, Tavarini S, Nuti S, De Gregorio E, Wack A. Intranasal Administration of CpG Induces a Rapid and Transient Cytokine Response Followed by Dendritic and Natural Killer Cell Activation and Recruitment in the Mouse Lung. J Innate Immun. 2010;2(2):144-59.
6. Kline JN, Waldschmidt TJ, Businga TR, Lemish JE, Weinstock JV, Thorne PS, Krieg AM. Modulation of airway inflammation by CpG oligodeoxynucleotides in a murine model of asthma. J Immunol.1998;160(6):2555-9.
7. Chang YS, Kim YK, Kwon HS, Park HW, Min KU, Kim YY, Cho SH. The effect of CpG-oligodeoxynucleotides with different backbone structures and 3' hexamericdeoxyriboguanosine run conjugation on the treatment of asthma in mice. J Korean Med Sci. 2009;24(5):860-6.
8. Ferraro M, Di Vincenzo S, Dino P, Bucchieri S, Cipollina C, Gjomarkaj M, Pace E. Budesonide, Aclidinium and Formoterol in combination limit inflammaging processes in bronchial epithelial cells exposed to cigarette smoke. Exp Gerontol.2019, 118:78-87.
9. Chen ZG, Zhang TT, Li HT, Chen FH, Zou XL, Ji JZ, Chen H. Neutralization of TSLP inhibits airway remodeling in a murine model of allergic asthma induced by chronic exposure to house dust mite. PLoS One*.* 2013, 8:e51268.
10. Luo Q, Lin J, Zhang L, Li H, Pan L. The anti-malaria drug artesunate inhibits cigarette smoke and ovalbumin concurrent exposure-induced airway inflammation and might reverse glucocorticoid insensitivity. Int Immunopharmacol*.*2015, 29:235-45.
11. Xu L, Sun WJ, Jia AJ, Qiu LL, Xiao B, Mu L, Li JM, Zhang XF, Wei Y, Peng C, Zhang DS, Xiang XD. MBD2 regulates differentiation and function of Th17 cells in neutrophils- dominant asthma via HIF-1alpha. J Inflamm (Lond)*.* 2018, 15:15.
12. Ramanathan SP, Helenius J, Stewart MP, Cattin CJ, Hyman AA, Muller DJ. Cdk1-dependent mitotic enrichment of cortical myosin II promotes cell rounding against confinement. Nat Cell Biol*.* 2015, 17:148-59.
13. Meng P, Chen ZG, Zhang TT, Liang ZZ, Zou XL, Yang HL, Li HT. IL-37 alleviates house dust mite-induced chronic allergic asthma by targeting TSLP through the NF-kappaB and ERK1/2 signaling pathways. Immunol Cell Biol*.*2019, 97:403-15.
